# The production of abnormal cells and Reed-Sternberg-like cells from normal lymphocytes.

**DOI:** 10.1038/bjc.1983.110

**Published:** 1983-05

**Authors:** A. E. Stuart

## Abstract

**Images:**


*Br. J. Cancer* (1983), **47**, 713–717

### Short Communication

### The production of abnormal cells and Reed-Sternberg-like cells from normal lymphocytes

A.E. Stuart

*University Department of Pathology, Royal Victoria Infirmary, Queen Victoria Road, Newcastle upon Tyne, NE1 4LP*

This preliminary study describes the presence of highly abnormal cells in cultures of human lymphocytes repeatedly stimulated with pokeweed mitogen. These cells are found with a low frequency which is increased several times after exposure to Hodgkin tissue. In these experiments Hodgkin tissue has been cultured in a glass vessel separated by a Millipore membrane from an outer flask containing peripheral blood mononuclear cells and a mitogen. The plan of the experiments has been to study autologous reactions between biopsy and blood samples from the same individual and allogeneic reactions between random samples.

Hodgkin tissue obtained at biopsy was divided into 2 parts, one for experimental use and the other for diagnosis. The biopsies were classified according to the Ann Arbor Convention. Normal thymus was obtained in fresh condition from the operating theatres. The Hodgkin tissue was used immediately, thymus and tissue from two cases of lymphoblastic lymphoma were stored in liquid nitrogen.

Normal samples of mononuclear cells were obtained from 2 regular blood donors (AES and SB) and 18 others. Further samples were taken from 6 patients with Hodgkin's disease at time of biopsy. The mononuclear cells were separated on Ficoll-Hypaque and cultured in the outer chamber at a concentration of $2.5 \times 10^7$ cells $ml^{-1}$.

The culture chamber comprised a 25 ml conical flask containing an inner tube of 1 cm diameter closed at the bottom end by a Millipore membrane of pore diameter 0.45 $\mu$m. The mononuclear cells were stimulated with pokeweed mitogen on days 0, 3 and 7 in a dose of 4 $\mu g\, ml^{-1}$. Cytocentrifuge specimens were taken daily, stained by Giemsa's method and examined for abnormal cells. Experience has shown that these usually appear within a restricted period and the quantitative data reported here refer only to Days 5, 6 and 7, with the exception of Case 6. Dissociated Hodgkin tissue was cultured in the inner chamber at a concentration of $10^7$ cells $ml^{-1}$ as were control cells when available from non-Hodgkin lymphoma and human thymus. In every experiment mononuclear cells treated with mitogen only were included as controls. All cells were cultured in RMPI 1640 with HEPES buffer, 10% foetal calf serum and standard supplements of penicillin and streptomycin.

The possibility of liberation of cytotoxic products from the inner chamber was tested by measuring trypan blue exclusion on lymphocytes in the outer chamber. Two sources of blood mononuclears and two preparations of Hodgkin tissue were used.

Cytospin preparations were stained for non-specific esterase using pararosaniline and alpha-naphthyl acetate according to Yam *et al.* (1971). Cell suspensions were treated with 4 monoclonal antibodies, DA6.231, OKT3, OKM1 and FMC1. DA6.231 (the kind gift of Dr. M. Steel) is prepared against the Burkitt line Daudi; it detects an epitope common to more than one set of DR molecules (Guy *et al.*, 1982). OKM1 (Ortho) is reactive with 78% of adherent mononuclear cells and 18% of the small non-adherent population (null cells). OKT3 reacts with 100% of peripheral T lymphocytes and 20% of thymocytes. FMC1 is a Flinders Medical Centre product which reacts with human B lymphoblastoid cell lines, with CLL cells and with peripheral blood B lymphocytes; it does not react with monocytes or T cells (Brooks *et al.*, 1980). Surface immunoglobulin was detected by the direct method using fluorescein-conjugated polyvalent anti-human immunoglobulin (Nordic). The preparations were examined by phase contrast and fluorescence microscopy.

The percentages of abnormal cells in proliferating lymphocytes exposed to autologous Hodgkin tissue are shown in Table I. The types of Hodgkin tissue included one case of lymphocyte predominance, two cases of nodular sclerosis and three cases of mixed cellularity. Case 5 yielded 10% of abnormal cells, Cases 3 and 6 gave lower values of 2.0 and 3.0% respectively. The control values when thymus and NHL were substituted for Hodgkin tissue never exceeded 0.8%. Cases 1, 2 and 4 gave 5.0, 2.8 and 6.0% abnormal cells with a maximum mitogen control figure on Case 4 of 1.0%.

Received 24 January 1983; accepted 14 February 1983.

© The Macmillan Press Ltd., 1983

**Table I** Percentages of abnormal cells in proliferating lymphocytes exposed to autologous Hodgkin tissue

| | *Case 1* | | | *Case 2* | | | *Case 3* | | | *Case 4* | | | *Case 5* | | | *Case 6* | | |
|---|---|---|---|---|---|---|---|---|---|---|---|---|---|---|---|---|---|---|
| | *Day* | | | *Day* | | | *Day* | | | *Day* | | | *Day* | | | *Day* | | |
| | *5* | *6* | *7* | *5* | *6* | *7* | *5* | *6* | *7* | *5* | *6* | *7* | *5* | *6* | *7* | *7* | *8* | *9* |
| Type of H.D. | Lymphocyte predominant | | | Nodular sclerosis | | | Nodular sclerosis | | | Mixed cell | | | Mixed cell | | | Mixed cell | | |
| Test culture | — | 5.0 | 1.7 | 2.8 | 1.0 | 1.4 | 1.6 | 2.0 | 1.5 | — | 6.0 | 1.0 | 10.0 | 4.0 | 1.0 | 0.5 | 3.0 | 1.0 |
| Mitogen control | — | 0.4 | 0.6 | 1.2 | 0.2 | 0.2 | 0.2 | 0.1 | 0.8 | — | 0.5 | 1.0 | 0.2 | 0.1 | 0.3 | 0.1 | 0.1 | 0.1 |
| Thymus | — | — | — | — | — | — | — | — | — | — | — | — | 0.1 | 0.2 | 0.2 | 0.2 | 0.4 | 0.1 |
| N.H.L. | — | — | — | — | — | — | 0.2 | 0.1 | 0.4 | — | — | — | 0.1 | 0.4 | 0.1 | 0.3 | 0.3 | 0.2 |

Allogeneic reactions between the blood donors SB and AES to 11 HD biopsies from different patients gave only one positive result. Reactions between 11 normal blood donors and a single source of Hodgkin tissue also gave only one positive result. These two positive reactions are given as percentages in Table II. Values of 6.0 and 8.0% were obtained with control values of 1.0% in cells exposed to mitogen alone.

The viability of cells in the outer chamber varied from 67–85% over Days 5, 6 and 7 and did not differ from the PWM control. No cytotoxic effect of HD tissue was thus observed.

The abnormal cells gave negative or a weak diffuse para-nuclear reaction when stained for non-specific esterase. Macrophages gave an intense reaction.

One hundred and twenty-three abnormal cells were directly observed, 19 out of 38 exhibited Ia-like antigen, 14 out of 35 reacted with OKT3, and 4 out of 17 expressed membrane Ig. Only 2 out of 29 reacted with OKM1 and of the very small number counted, 0 out of 4 reacted with FMC1.

All forms of morphological abnormality were found in control cultures although numbers were conspicuously increased in the autologous group. Four different types of nuclear pattern were seen:

(a) Large binucleate cells with abundant pale blue cytoplasm devoid of granules. The nuclei are round with a compact reticulate pattern, nucleoli being inconspicuous or absent. These cells resemble reactive binucleate cells of plasmacytoid type found in some chronic inflammatory conditions.

(b) Nuclear pouches were seen projecting from the main body of the nucleus (Figure 1). These commence as tiny nodules, usually solitary, and are attached by a narrow stalk to the main nucleus. Larger forms are like sprouting beans and resemble a second nucleus.

(c) Binucleate cells, some of "mirror-image" type (Figure 2) and ranging in size from 40–80 $\mu$m were observed. The nuclei usually had a homogenous appearance and large eosinophilic nucleoli were commonly present. An uncommon form has been noted in which the faint outlines of what appear to be spherical nuclei can be discerned within the substance of the parent nuclei (Figure 3).

(d) Large multinucleated cells with convoluted nuclei of cottage loaf appearance (Figure 4). The enormous nucleus is twisted and convolved in serpiginous folds. Some of these bizarre cells contain isolated blocks of nuclear material lying free in the cytoplasm (Figure 5). Sometimes these segregated nuclei are connected with the main nucleus by thin threads of chromatin (Figure 6).

**Table II** Percentages of abnormal cells found in two positive heterologous reactions

| | *D (blood donor) vs R (biopsy donor)* | | | | | *S.B. (blood donor) vs B (biopsy donor)* | | |
|---|---|---|---|---|---|---|---|---|
| Days | 8 | 10 | 11 | 12 | 15 | 5 | 7 | 10 |
| Percentage abnormal cells | 4.0 | 8.0 | 5.0 | 2.0 | 1.0 | 3.0 | 6.0 | 4.0 |
| Control | ← | | <1.0 | | → | ← | <1.0 | → |

**Figure 1** "Bean sprout" type of abnormality. × 1200.

**Figure 3** Symmetrical nuclei with internal contours of individual spherical nuclei. × 1200.

**Figure 2** Reed-Sternberg-like cell. × 1200.

(e) Groups of small particles staining purple with Giemsa have been noted lying very close to the cell nucleus.

The number of abnormal cells, regularly found in mitogen-stimulated cultures of blood cells from normal donors, is low, unlike that found following exposure to Hodgkin tissue where the increase can be as great as 10-fold. Is this a non-specific effect mediated by products of cell disintegration within the inner tube? Substitution of normal thymus and lymphomatous tissue other than Hodgkin's disease did not enhance the number of abnormal cells. The majority of experiments involving allogeneic interactions failed to produce atypical cells and this strengthens the argument that the effect depends both on the presence of Hodgkin tissue and on an autologous environment. A substitute for mitogen-stimulated peripheral blood mononuclear cells would be useful but unfortunately gross nuclear abnormalities are found in many human cell lines making them unsuitable target cells for the detection of nuclear changes.

The finding of increased numbers of abnormal cells could be due to an increased number of abnormal cells formed, or to loss of normal cells from the system due to cytotoxicity. No differences were found in the viability of test and control

**Figure 4** Example of a convoluted nucleus. × 1200.

**Figure 5** Example of gross nuclear dysgenesis. × 1200.

**Figure 6** Abnormal cell with bridging thread between nuclear masses. × 1200.

cultures and therefore it seems that the increase observed is genuine.

These cells could be either lymphocytic or monocytic. However, their lack of non-specific esterase and failure to react with the monoclonal antibody OKM1 indicates that a monocytic origin is unlikely. All exhibited Ia-like antigen but this does not help in deciding their nature since a proportion of activated T cells and monocytes express this determinant. The positive results with OKT3 are certainly consistent with an origin of some from T cells and the expression of surface immunoglobulin by a minority is compatible with a B cell derivation. Clearly further studies with a much wider range of antisera are required to answer the question whether these unusual cells belong to a particular lymphocyte subset.

The resemblance of some abnormal cells to Reed-Sternberg cells is striking. Their large size, ample and glassy cytoplasm, lobulated nuclei and prominent nucleoli are indistinguishable from the true Reed-Sternberg cells found in paraffin sections or cytospin preparations of Hodgkin tissue. The finding of these cells in normal cultures and their augmentation by exposure to Hodgkin tissue is consistent with the hypothesis that normal persons have the capacity to produce grossly abnormal lymphoid cells which are usually suppressed, whereas in Hodgkin's disease some factor in the diseased lymph nodes facilitates their production and persistence.

The valuable help of Dr. E. Jackson and Ms. V. Brydon is gratefully acknowledged, as is the assistance of Ms. B. Kennedy who typed the manuscript. This work has been assisted by the North of England Cancer Research Campaign.

### References

BROOKS, D.A., BECKMAN, I., BRADLEY, J., MCNAMARA, P.J., THOMAS, M.E. & ZOLA, H. (1980). Human lymphocyte markers defined by antibodies derived from somatic cell hybrids. *Clin. Exp. Immunol.*, **39**, 477.

GUY, K., VAN HEYNINGEN, V. COHEN, B.B., DEAN, D.L., CRICHTON, D. & STEEL, C.M. (1982). Subsets of human D locus products identified by a series of monoclonal antibodies. In *Protides of The Biological Fluids 29th Colloquium 1981* Oxford: Pergamon Press.

YAM, L.T., LI, C.Y. & CROSBY, W.H. (1971). Cytochemical identification of monocytes and granulocytes. *Am. J. Clin. Pathol.*, **55**, 283.


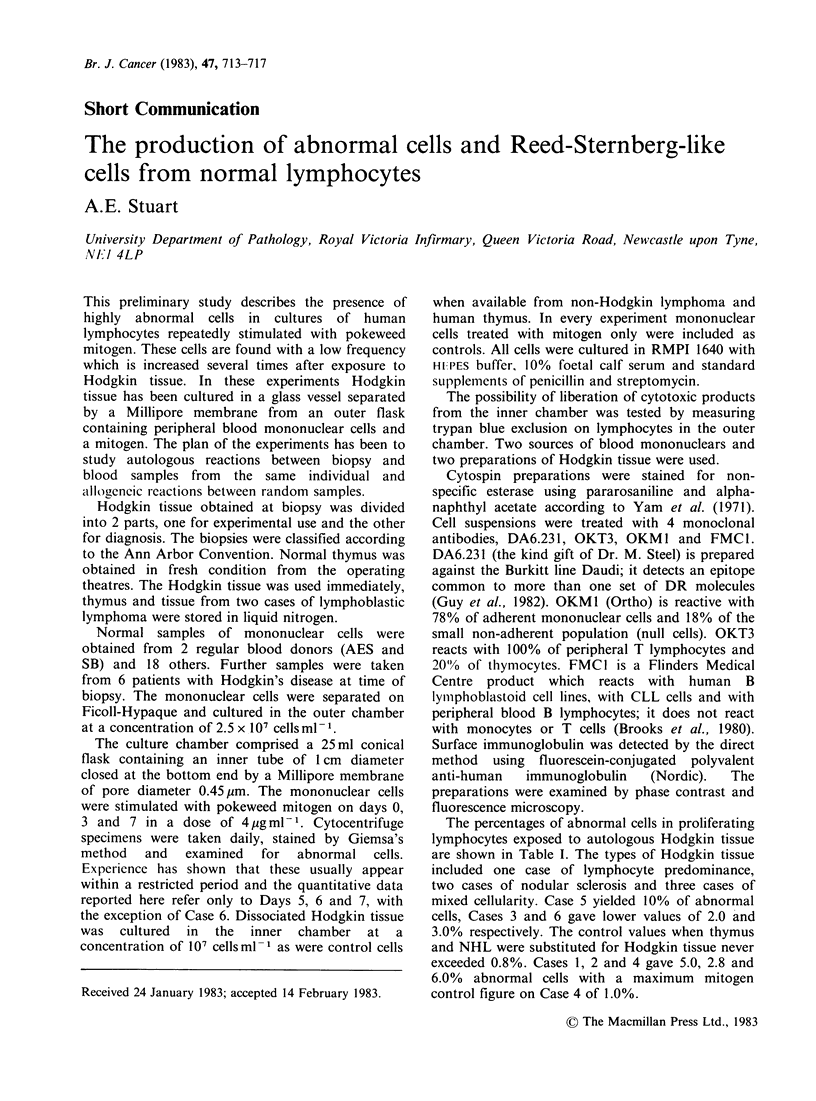

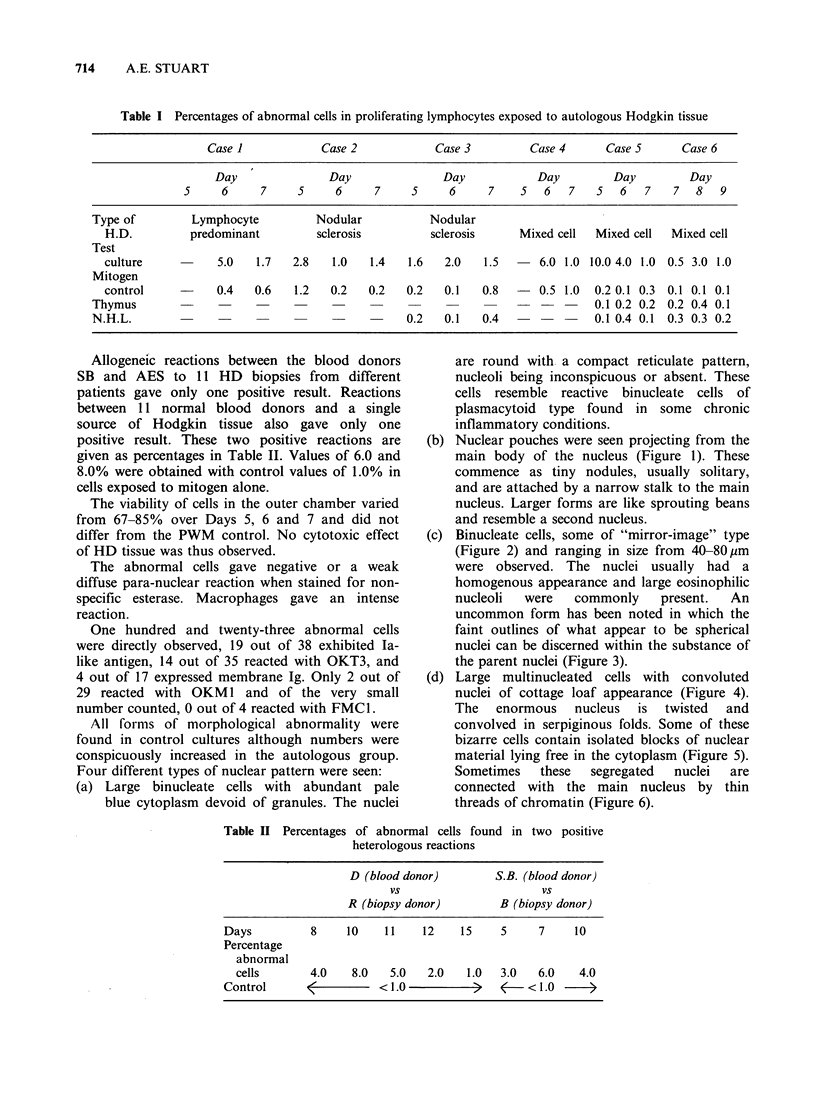

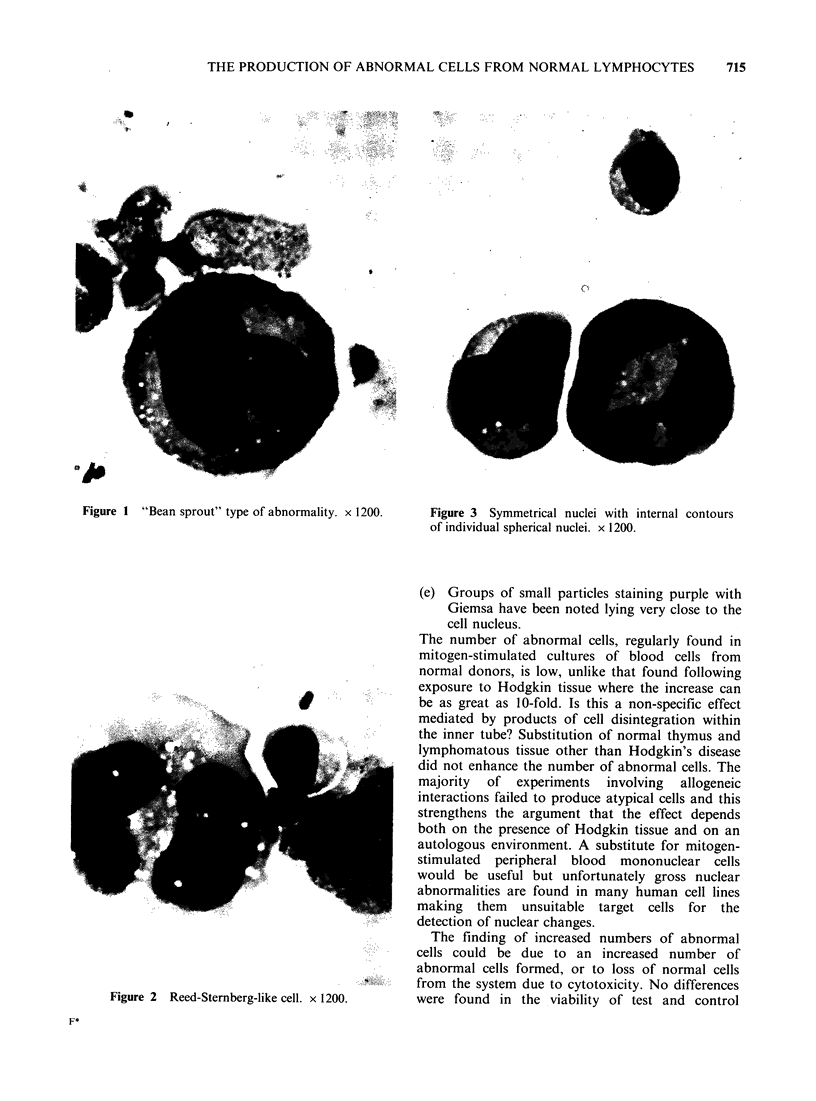

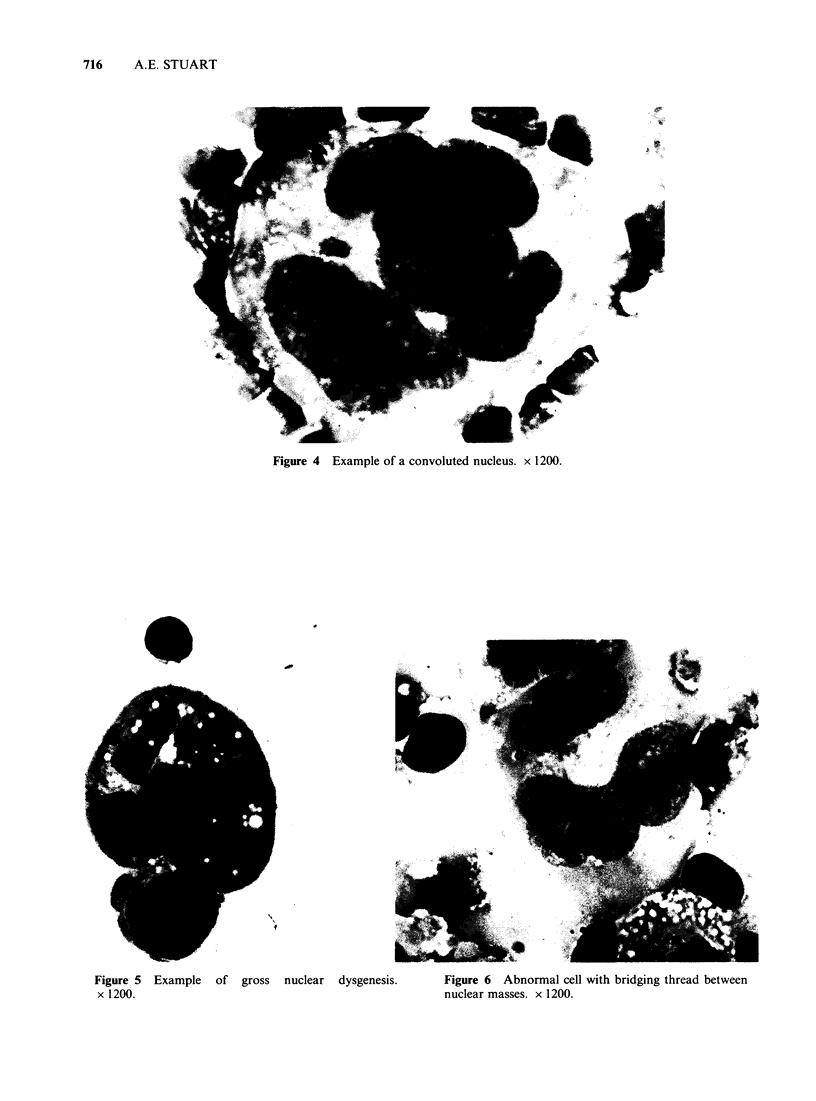

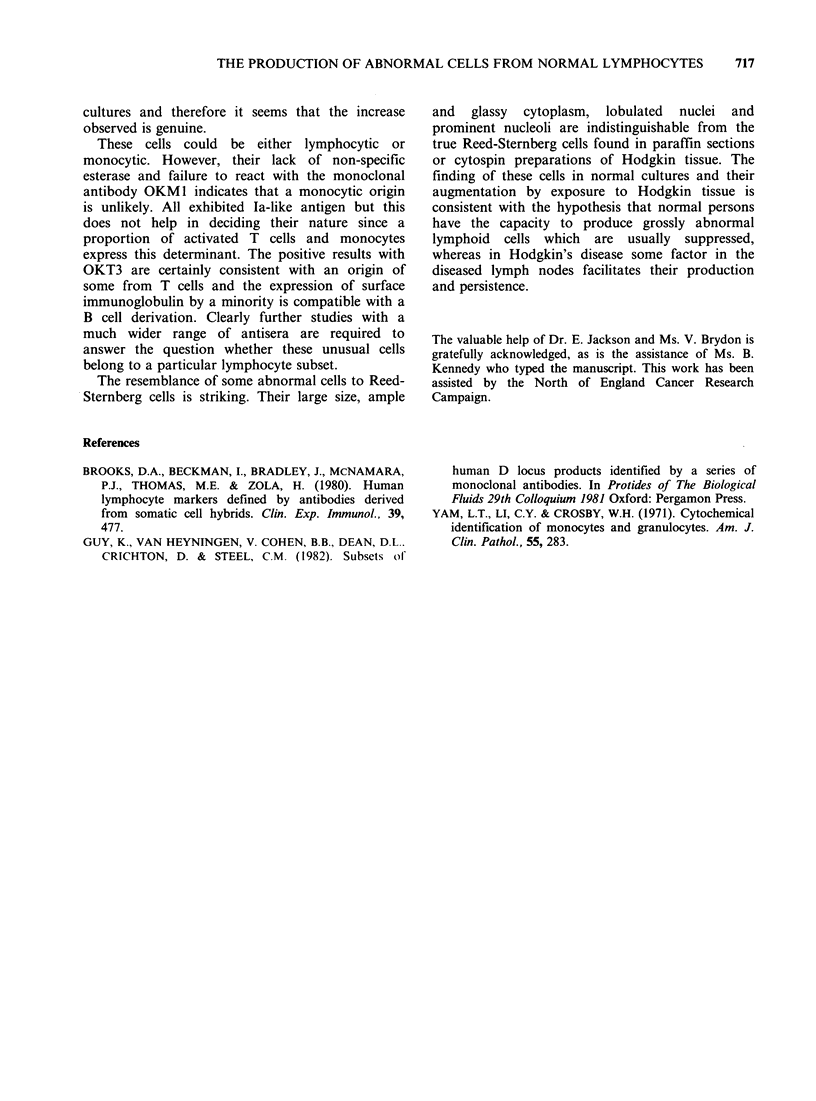


## References

[OCR_00327] Brooks D. A., Beckman I., Bradley J., McNamara P. J., Thomas M. E., Zola H. (1980). Human lymphocyte markers defined by antibodies derived from somatic cell hybrids. I. A hybridoma secreting antibody against a marker specific for human B lymphocytes.. Clin Exp Immunol.

[OCR_00342] Yam L. T., Li C. Y., Crosby W. H. (1971). Cytochemical identification of monocytes and granulocytes.. Am J Clin Pathol.

